# Compositional analyses of the associations between sedentary time, different intensities of physical activity, and cardiometabolic biomarkers among children and youth from the United States

**DOI:** 10.1371/journal.pone.0220009

**Published:** 2019-07-22

**Authors:** Valerie Carson, Mark S. Tremblay, Jean-Philippe Chaput, Duncan McGregor, Sebastien Chastin

**Affiliations:** 1 Faculty of Kinesiology, Sport and Recreation, University of Alberta, Edmonton, AB, Canada; 2 Healthy Active Living and Obesity Research Group, Children’s Hospital of Eastern Ontario Research Institute, Ottawa, ON, Canada; 3 School of Health and Social Care, Glasgow Caledonian University, Glasgow, United Kingdom; 4 Department of Movement and Sport Science, Ghent University, Ghent, Belgium; Linneaus University, SWEDEN

## Abstract

**Introduction:**

Compositional data analysis is one appropriate method for co-dependent data, even when data are collected for a subdivision of the 24-hour period, such as the waking day. Objectives were to use compositional analyses to examine the combined and relative associations of sedentary time (ST), light-intensity physical activity (LPA), moderate-intensity physical activity (MPA), and vigorous-intensity physical activity (VPA) with cardiometabolic biomarkers in a representative sample of children and youth.

**Methods:**

This cross-sectional study included 2544 participants aged 6–17 years from the 2003–2006 United States National Health and Nutrition Examination Survey. ST (<100 counts per minute), LPA (100 counts per minute to <4 METs; Freedson age-specific equation), MPA (4 to <7 METs), and VPA (≥7 METs) were accelerometer-derived. Cardiometabolic biomarkers included waist circumference, body mass index (BMI) z-score, HDL-cholesterol, C-reactive protein, and blood pressure. Triglycerides, glucose, insulin, and LDL-cholesterol were measured in a fasting sub-sample of adolescents (n = 670). Compositional linear regression models were conducted.

**Results:**

The composition of ST, LPA, MPA, and VPA was significantly associated with BMI z-score, log waist circumference, systolic and diastolic blood pressure, HDL-cholesterol, and log plasma glucose (variance explained: 1–29%). Relative to the other three behaviors, VPA was negatively associated with BMI z-score (γVPA = -0.206, p = 0.005) and waist circumference (γVPA = -0.03, p = 0.001). Conversely, ST was positively associated with waist circumference (γST = 0.029, p = 0.013). ST and VPA were also positively associated with diastolic blood pressure (γST = 2.700, p = 0.018; γVPA = 1.246, p = 0.038), relative to the other behaviors, whereas negative associations were observed for LPA (γLPA = -2.892, p = 0.026). Finally, VPA was positively associated with HDL-cholesterol, relative to other behaviors (γVPA = 0.058, p<0.001).

**Conclusions:**

The ST and physical activity composition appears important for many aspects of cardiometabolic health in children and youth. Compositions with more time in higher-intensity activities may be better for some aspects of cardiometabolic health.

## Introduction

Children and youth can engage in behaviors of various intensities throughout the day. The role of moderate- to vigorous-intensity physical activity (MVPA) in the promotion of health and the prevention of disease in children and youth has been the focus of numerous studies.[1] Though there has been less research that has considered moderate-intensity physical activity (MPA) and vigorous-intensity physical activity (VPA) as separate behaviors.[1] At the other end of the intensity spectrum, there has been extraordinary growth in sedentary behavior research over the past decade,[2,3] given it makes up a substantial portion (~50–60%) of the waking day among children and youth.[4,5] Light-intensity physical activity (LPA), which also makes up a large proportion of the waking day (~30%),[4] has also been the focus of increasing research.[1]

The increasing research on sedentary behavior and LPA has resulted in growing discussions and debate on whether sedentary behavior predicts health indicators independent of physical activity.[6] A number of studies in children and youth have adjusted for MVPA in regression models examining the association between sedentary behavior and health indicators.[2] However, LPA is typically not adjusted for because of methodological issues. Specifically, LPA is highly correlated with sedentary behavior and therefore including both variables in a regression model results in collinearity issues.[6] In fact, the total time spent in sedentary behavior (ST), LPA, MPA, and VPA during the waking day is finite and perfectly collinear.[6] These methodological issues make it challenging to understand the collective impact of these behaviors on health and how best to intervene.

Compositional analysis is one appropriate method for finite and co-dependent data but has only recently been used in the physical activity field.[7,8] This method has primarily been used in samples of children and youth with 24-hour data (i.e., ST, LPA, MPA, VPA, sleep duration) and findings across studies indicate that the composition of behaviors within the 24-hour period is important for health.[8–11] However, some studies are only interested in specific settings to inform targeted interventions (e.g., school)[12] and some studies have not collected 24-hour data. For example, the National Health and Nutrition Examination Survey (NHANES) has previously used a waking day protocol for accelerometer data with no corresponding sleep duration data in participants <16 years old. Data collected for a subdivision of the 24-hour period (e.g., waking day) still represents a finite amount of time making compositional analysis an appropriate method.[7] Therefore, the objectives of this study were to use compositional analyses to examine: 1) the combined associations of ST, LPA, MPA, and VPA with cardiometabolic biomarkers; and (2) the association of time spent in ST, LPA, MPA, and VPA with cardiometabolic biomarkers relative to the time spent in the other three behaviors in a representative sample of children and youth from the NHANES.

## Materials and methods

### Participants

Participants were 6–17 year olds of the 2003–2004 and 2005–2006 cycles of the NHANES. This large survey uses a repeated cross-sectional design and involves a complex, four-stage sampling procedure to capture a nationally representative sample of the resident civilian noninstitutionalized United States population.[13] Additional details regarding NHANES are available elsewhere.[13–15] Across the two cycles, a total of 5,607 participants aged 6–17 years were enrolled, and a total of 4,672 wore an accelerometer making them eligible for this study. Ethics approval was obtained from the National Centre for Health Statistics Research Ethics Review Board (Protocol # 98–12; Protocol # 2005–06). Written informed consent was obtained from the parents/guardians of all participants. Consent/assent was also obtained from those 7–17 years.[15]

### Sedentary time and physical activity

ST and physical activity (PA) were measured with waist-worn uniaxial ActiGraph 7164 accelerometers (ActiGraph, Ft. Walton Beach, FL). Participants were given the accelerometer at a mobile examination center as part of their physical exam and asked to wear it for 7 consecutive days apart from sleeping and water-based activities.[14] After data download, biologically implausible values were excluded by NHANES staff.[14] Data were collected in one minute epochs, and non-wear time was defined as ≥60 consecutive minutes of zero counts, with allowance for 1–2 minutes of counts between zero and 100.[16,17] To be included in the analyses, participants had to have ≥10 hours of wear time per day for ≥4 days.[16,17] ST was defined as <100 counts per minute (cpm), [18–20] LPA as 100 to <4 metabolic equivalents (METs) according to Freedson’s age-specific regression equation, [21] MPA as 4 to <7 METs, and VPA as ≥7 METs. These MET values align with moderate walking and vigorous running activities identified across child and adolescent age groups in the Youth Compendium of Physical Activities. [22] Average minutes per day of ST, LPA, MPA, and VPA were calculated through a series of steps to account for the fact that waking day data will fluctuate between days for a participant and between participants. First, geometric means of each intensity were calculated across valid days for each participant. Any daily values equaling zero minutes were replaced with 0.5 minutes prior to calculating the geometric mean. The vast majority of days (99.6%) with zero minutes had zero minutes for VPA. Second, wear time was calculated for each participant by adding up the minutes per day in each intensity. Third, the proportion of time spent at each intensity during wear time was calculated for each participant by dividing the geometric mean of each intensity by wear time. Finally, to aid interpretation, the proportions were converted in minutes, by multiplying the proportion of time spent at each intensity by the sample wear time mean.

### Cardiometabolic biomarkers

Cardiometabolic biomarkers were measured by trained personnel at the mobile examination center. Height, weight, waist circumference, HDL cholesterol, and C-reactive protein (CRP) were measured in the full analytical sample. Age- and sex-specific body mass index (BMI) z-scores were calculated based on the Centers for Disease Control and Prevention growth charts.[23] Waist circumference was measured at the level of the iliac crest.[24] HDL cholesterol (Roche/Boehringer-Mannheim Diagnostics direct HDL method) and CRP (nephelometry method) were measured from venous blood samples. [14] Systolic and diastolic blood pressure were measured manually three to four consecutive times on the right arm while seated in participants ≥8 years.[14,25] The average blood pressure across the repeated measurements was calculated. Additionally, LDL cholesterol (calculated using the Friedewald calculation from measured values of total cholesterol, triglycerides, and HDL-cholesterol), triglycerides (Hitachi 704 method in 2003–2004 and the Hitachi 717/912 method in 2005–2006), plasma glucose (enzyme hexokinase method in 2003–2004 and the hexokinase-mediated reaction in 2005–2006), and insulin (two-site immunoenzymometric assay method in 2003–2004 and the human insulin immunoassay in 2005–2006) were measured from venous blood samples in a sub-sample of participants (≥12 years) who attended the morning examination and provided fasting blood samples. [14] As recommended, correction equations were applied to plasma glucose and insulin to account for the different methods between cycles. [14] Only participants who reported fasting for ≥8 hours were included in the fasting sub-sample analyses. [14] Detailed descriptions of the procedures and methods used to measure each cardiometabolic biomarker are available on the NHANES website. [14]

### Covariates

Age, sex, race/ethnicity, socioeconomic status (SES), smoking, total dietary intake, saturated fat, and sodium intake were considered as covariates based on data availability and previous ST and PA research.[1,2] Race/ethnicity was classified into four groups (non-Hispanic White, non-Hispanic Black, Mexican American, other). SES was estimated using a poverty income ratio (a ratio of family income to poverty threshold). [14] Smoking was assessed by asking participants ≥12 years if they had previously tried cigarette smoking (yes or no). Those <12 years were categorized as no. Total dietary intake, saturated fat and sodium intake were derived from a 24-hour dietary recall. Saturated fat (<10% or ≥10% of total calories) and sodium (<2300 or ≥2300 mg/day) were dichotomized based on dietary guidelines in the United States.[26]

### Statistical analysis

Analyses were conducted in 2018/2019 using SAS version 9.4 (SAS Institute Inc., Cary, NC) and accounted for the complex design and sample weights of NHANES. Sample weights were re-weighted for the full analytical and fasting sub-sample based on missing data to achieve a representative sample. Traditional descriptive statistics were calculated for participant characteristics. Compositional descriptive statistics, including compositional geometric means (central tendency), a variation matrix (dispersion), and geometric mean bar plots (relative behavioral profiles for select cardiometabolic biomarkers) were calculated for ST and PA variables.[7] For the variation matrix, a value closer to zero indicates higher co-dependence between two behaviors.[7] The compositional geometric mean bar plots were calculated for BMI z-score, waist circumference, diastolic blood pressure, HDL-cholesterol, and glucose to capture different aspects of cardiometabolic health risk. Quartiles were used to create sub-groups for all cardiometabolic biomarkers with the exception of BMI where sub-groups included: underweight (<5^th^ percentile), normal or healthy weight (5^th^ to <85^th^ percentile), overweight (85^th^ to <95^th^ percentile), and obese (≥95^th^ percentile).[27]

To address objective one of the paper, four compositional linear regression models were conducted for each cardiometabolic biomarker sequentially rotating the sequence of ST, LPA, MPA and VPA and entering the composition of variables into the model via an isometric log-ratio transformation. The fluctuations in waking day data are accounted for in these analyses based on the calculation of average minutes per day of ST, LPA, MPA, and VPA described in the methods section. It is also important to note that the isometric log-ratio transformation is scale invariant so the proportion of time spent at each intensity based on the geometric means can also be used in these models and will produce the same results. Model p-values and R^2^ coefficients were obtained from linear regression models not adjusted by covariates and were used to determine whether there was a statistically significant association between the composition of ST and PA variables and each cardiometabolic biomarker, and the proportion of the variance explained by the composition. Model p-values and R^2^ coefficients are identical across the four regression models. After examining residuals, waist circumference, CRP, LDL-cholesterol, triglycerides, glucose and insulin, were log-transformed in line with the assumption of normality for regression models.

To address objective two of the paper, the first coefficient and the corresponding p-value for each of the four linear regression models were used to determine whether the time spent in each behavior was significantly associated with each cardiometabolic biomarker relative to the time spent in the other three behaviors. All models adjusted for age, sex, race/ethnicity, SES, smoking, total energy intake, sodium, and saturated fat. Additional analyses further adjusted for waist circumference, apart from waist circumference and BMI z-score models. All compositional analyses methods used have been outlined in detail in previous studies.[7,9,28] Statistical significance was set a priori at p<0.05.

## Results

Of the eligible 4,672 participants, 3,173 had valid accelerometer data, and of those participants, 2,544 had complete cardiometabolic biomarkers and covariate data in the full analytical sample. For blood pressure, 2,178 (diastolic) to 2,196 (systolic) participants aged 8–17 years had complete data. For the fasting sub-sample, 2,923 participants aged 12–17 years were eligible and 1,940 had valid accelerometer data. Of those participants, 670 had complete cardiometabolic biomarkers and covariate data. Participant characteristics for the total sample (n = 5,607), full analytical sample (n = 2,544), and fasting sub-sample (n = 670) are presented in Table 1. Through the process of reweighting the sample weights due to missing data, the full analytical sample and fasting sub-sample (for sex and race/ethnicity) closely aligned with the total sample.

**Table 1:** Weighted participant characteristics of the 2003/04 and 2005/06 cycles of the NHANES.

Variables	Total sample (n = 5607)	Full analytical sample (n = 2544)	Fasting sub-sample (n = 670)
Age (years)	11.2 (8.1–14.1)	11.9 (8.8–14.3)	13.8 (12.5–15.3)
Sex (%)			
Male	51.1	51.1	50.9
Female	48.9	48.9	49.1
Race (%)			
Non-Hispanic White	60.8	61.5	63.1
Non-Hispanic Black	14.8	15.0	15.0
Mexican-American	12.4	12.2	11.3
Other	11.9	11.3	10.7
Poverty income ratio	-	2.5 (1.2–4.1)	2.8 (1.4–4.3)
Ever tried smoking (%)			
Yes	-	16.9	28.4
No	-	83.1	71.6
Total energy intake (kcal)	-	2029.8 (1571.9–2574.0)	2138.5 (1637.0–2751.1)
Sodium Intake (%)			
≤2300 mg/day	-	25.8	25.0
>2300 mg/day	-	74.2	75.0
Saturated Fat (%)			
≤10% of total calories	-	30.3	37.1
>10% of total calories	-	69.7	62.9
Cardiometabolic Biomarkers			
BMI z-score	-	0.5 (-0.3–1.3)	-
Waist Circumference (cm)	-	71.3 (70.5–72.0)	-
Systolic Blood Pressure (mmHg; n = 2196)	-	105.9 (98.9–112.1)	-
Diastolic Blood Pressure (mmHg; n = 2178)	-	58.6 (50.9–65.4)	-
HDL-Cholesterol (mmol/L)	-	1.4 (1.2–1.6)	-
C-reactive Protein (mg/L)	-	0.3 (0.1–1.0)	-
LDL-Cholesterol (mmol/L)	-	-	2.2 (1.8–2.6)
Triglycerides (mmol/L)	-		0.8 (0.6–1.1)
Plasma Glucose (mmol/L)	-	-	5.0 (4.8–5.3)
Insulin (pmol/L)	-	-	54.5 (37.3–79.5)

BMI, body mass index; HDL, High-density lipoprotein cholesterol; LDL, Low-density lipoprotein cholesterol; NHANES, National Health and Nutrition Examination Survey.

Data presented as median (Inter-quartile range) for continuous variables and percentage for categorical variables.

The geometric means and corresponding % of wear time for the ST and PA variables are presented in Table 2. In the full analytical sample, participants spent the majority of their waking day in either ST or LPA, with less than 4% of their time spent in MPA and VPA. The pair-wise log-ratio variation matrix is presented in Table 3. ST and LPA had the highest co-dependence (0.00025), followed by MPA and VPA (0.00042). The lowest co-dependence was seen between MPA and ST (0.00205) and VPA and ST (0.00167).

**Table 2:** Geometric means for ST and PA in minutes/day and corresponding percentage of wear time.

	Full analytical sample (6–17 years)		Fasting sub-sample (12–17 years)	
	n = 2544		n = 670	
	Minutes/day	% of wear time	Minutes/day	% of wear time
ST	402	51.8	463	58.3
LPA	347	44.7	318	40.0
MPA	24	3.1	12	1.5
VPA	3	0.4	1	0.1

LPA, light-intensity physical activity; MPA, moderate-intensity physical activity; PA, physical activity; ST, sedentary time; VPA, vigorous-intensity physical activity.

PA and ST variables have been normalized on average wear time.

**Table 3:** Pair-wise log-ratio variation matrix for ST and PA in the full analytical sample (n = 2544).

	ST	LPA	MPA	VPA
ST	0	0.00025	0.00205	0.00167
LPA	0.00025	0	0.00161	0.00125
MPA	0.00205	0.00161	0	0.00042
VPA	0.00167	0.00125	0.00042	0

LPA, light-intensity physical activity; MPA, moderate-intensity physical activity; PA, physical activity; ST, sedentary time; VPA, vigorous-intensity physical activity.

**Fig 1:**
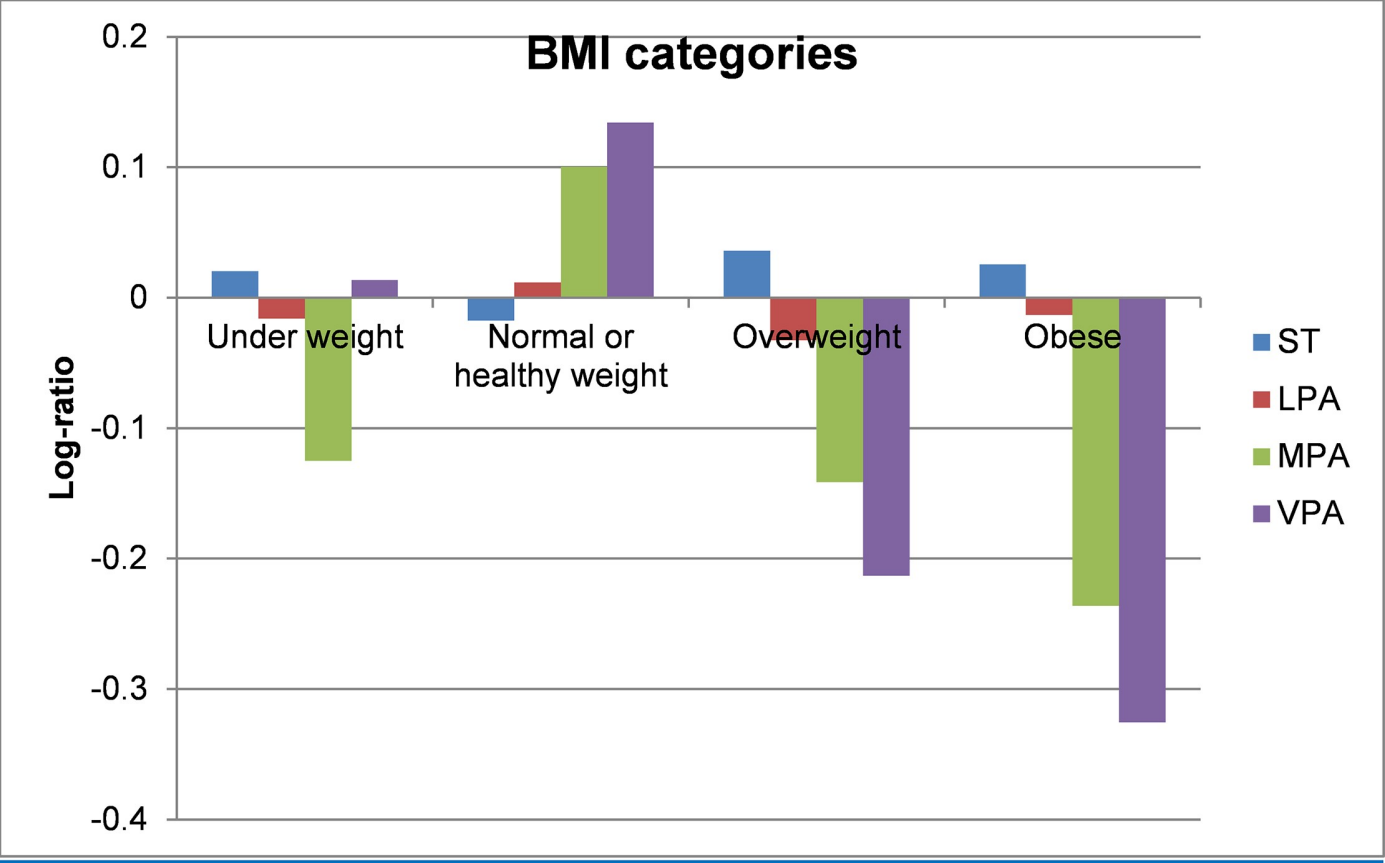
Compositional geometric mean bar plots comparing the compositional mean of the entire sample with the compositional mean of underweight, normal weight, overweight, and obese subgroups for sedentary time (ST), light-intensity physical activity (LPA), moderate-intensity physical activity (MPA), and vigorous-intensity physical activity (VPA). BMI = body mass index.

**Fig 2:**
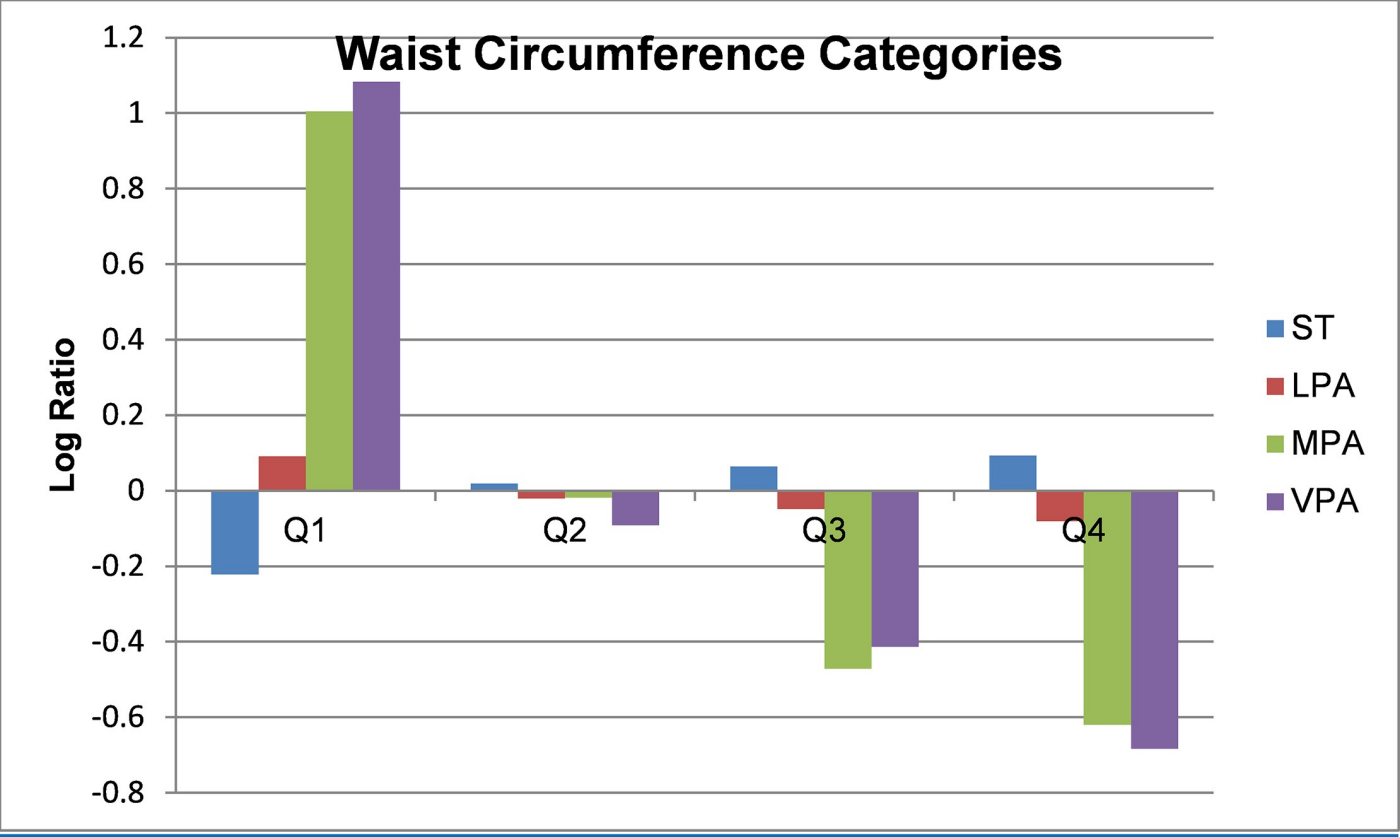
Compositional geometric mean bar plots the compositional mean of the entire sample with the compositional mean of waist circumference quartiles subgroups for sedentary time (ST), light-intensity physical activity (LPA), moderate-intensity physical activity (MPA), and vigorous-intensity physical activity (VPA).

**Fig 3:**
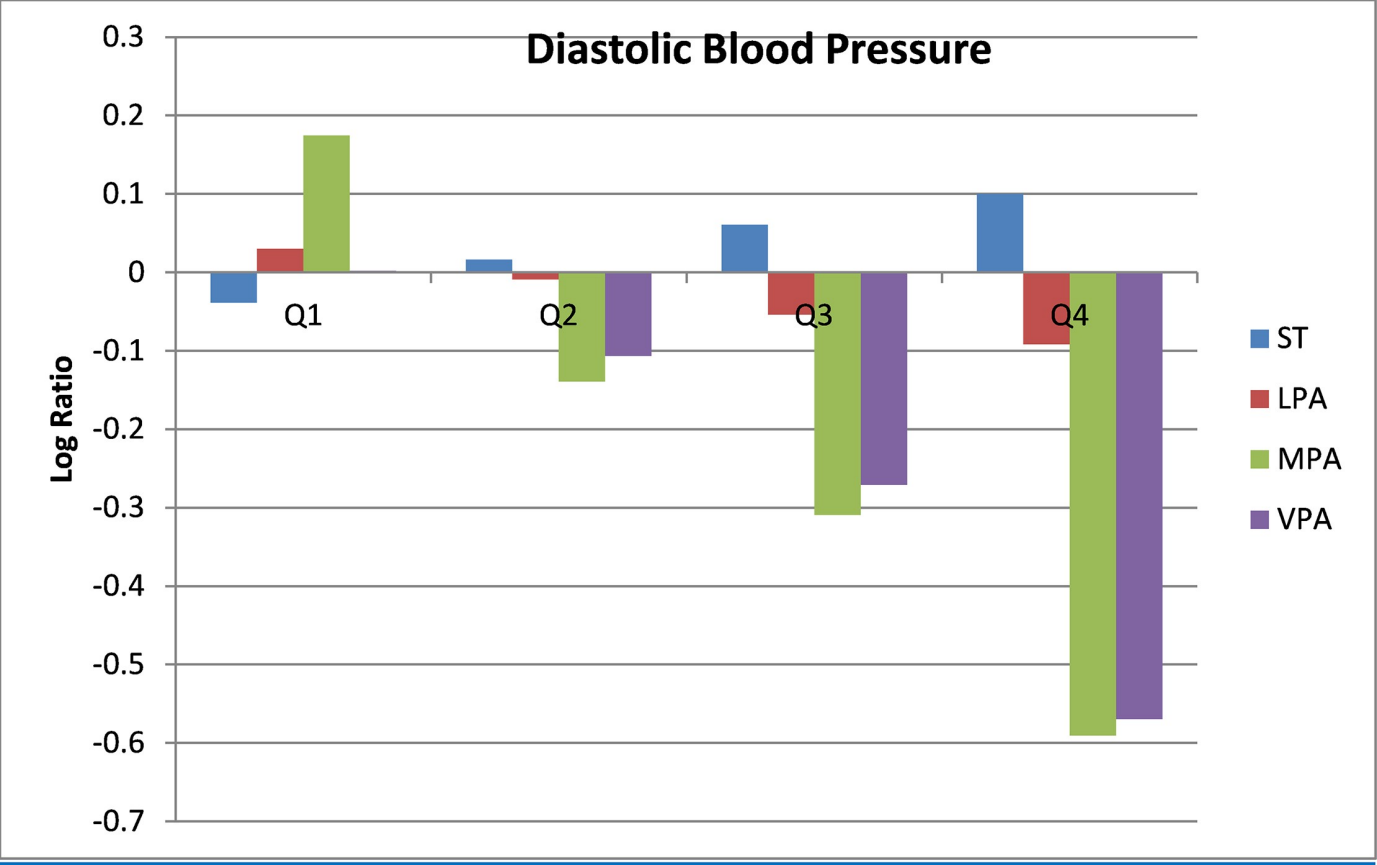
Compositional geometric mean bar plots comparing the compositional mean of the entire sample with the compositional mean of diastolic blood pressure quartiles subgroups for sedentary time (ST), light-intensity physical activity (LPA), moderate-intensity physical activity (MPA), and vigorous-intensity physical activity (VPA).

**Fig 4:**
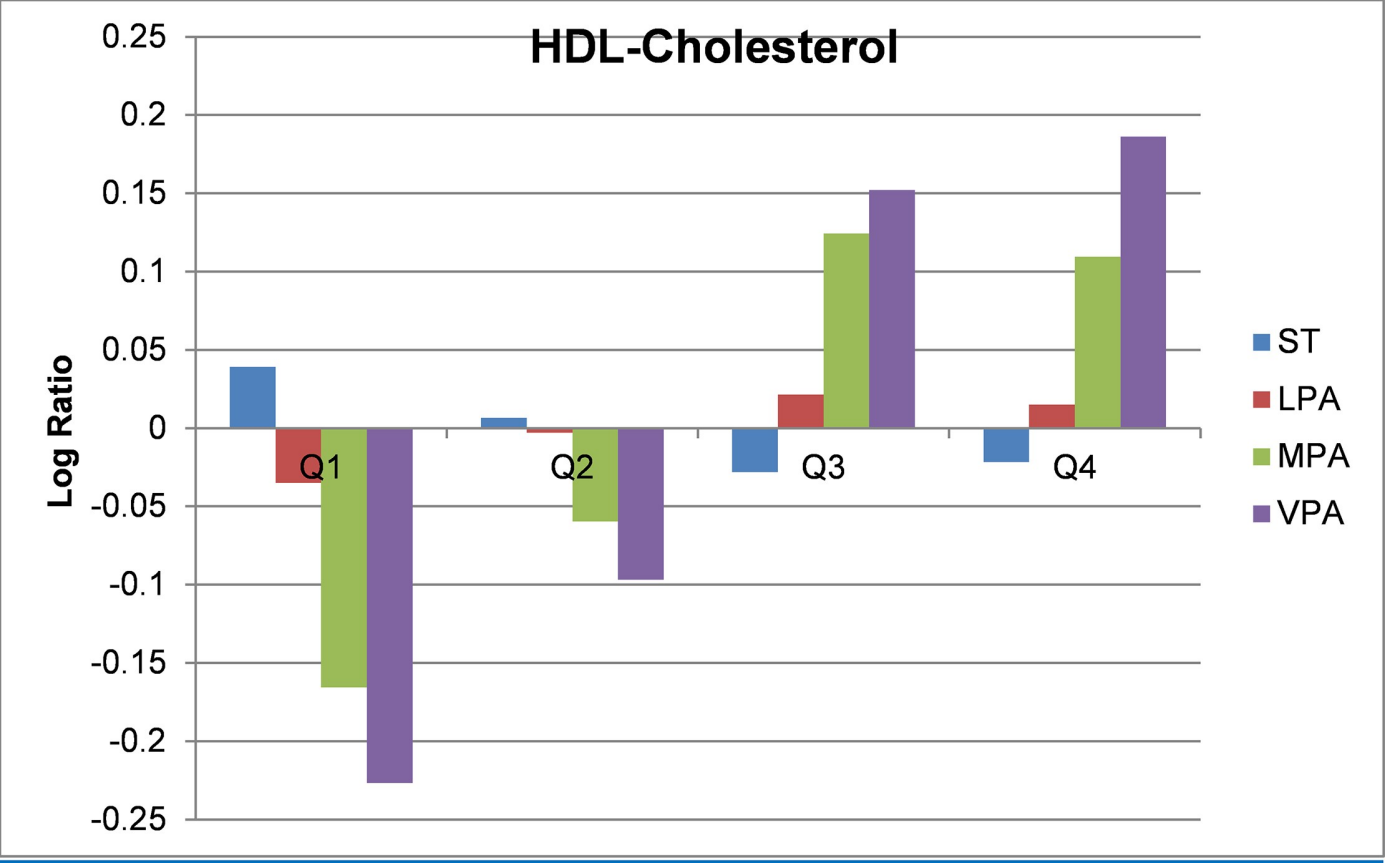
Compositional geometric mean bar plots comparing the compositional mean of the entire sample with the compositional mean of HDL-cholesterol quartiles subgroups for sedentary time (ST), light-intensity physical activity (LPA), moderate-intensity physical activity (MPA), and vigorous-intensity physical activity (VPA); HDL = High-density lipoprotein cholesterol.

Compositional geometric mean bar plots for select cardiometabolic biomarkers are presented in Figs 1–4. For BMI categories, the proportion of ST was lower and the proportion of time spent in all intensities of PA were higher in the normal or healthy weight group relative to the entire sample. The overweight and obese groups had higher ST and lower MPA and VPA relative to the entire sample, with the lowest MPA and VPA being observed in the obese group. Participants in the lowest quartile of waist circumference and diastolic blood pressure (Q1) tended to have lower ST and higher LPA, MPA, and VPA relative the entire sample. For the most part, the opposite was observed for participants in the highest quartile (Q4). The same pattern but reversed was observed for HDL-cholesterol.

The results of the four compositional linear regression models are shown in Table 4. The composition of ST and PA variables were significantly associated with BMI z-score, log waist circumference, systolic and diastolic blood pressure, HDL-cholesterol, and log plasma glucose (model p-value: 0.004 to <0.001). The proportion of the variance explained by the composition for those cardiometabolic biomarkers ranged from 1 to 29%.

**Table 4:** Compositional linear regression for the associations between ST, LPA, MPA, and VPA and cardiometabolic biomarkers.

Cardiometabolic biomarkers	Model p-value	Model R-square	γ_ST_	p-value	_γLPA_	p-value	_γMPA_	p-value	_γVPA_	p-value
Full Analytical Sample (n = 2544)										
BMI z-score	**<0.001**	**0.013**	0.111	0.279	0.041	0.754	0.054	0.390	**-0.206**	**0.005**
Log waist circumference	**<0.001**	**0.290**	**0.029**	**0.013**	-0.006	0.708	0.010	0.325	**-0.033**	**0.001**
Systolic blood pressure (n = 2178)	**<0.001**	**0.059**	0.952	0.234	0.196	0.857	-0.454	0.321	0.689	0.215
Diastolic blood pressure (n = 2178)	**<0.001**	**0.070**	**2.700**	**0.018**	**-2.892**	**0.026**	-1.051	0.095	**1.246**	**0.038**
HDL-cholesterol	**0.002**	**0.011**	-0.038	0.097	0.014	0.554	**-0.035**	**0.034**^a^	**0.058**	**<0.001**
Log C-Reactive Protein	0.058	0.009	0.097	0.478	0.001	0.996	0.055	0.524	-0.153	0.094
Fasting Sub-sample (n = 670)										
Log LDL-cholesterol	0.795	0.003	-0.018	0.684	0.011	0.856	-0.008	0.757	0.015	0.564
Log triglycerides	0.513	0.006	0.015	0.839	0.022	0.778	0.003	0.938	-0.041	0.311
Log plasma glucose	**0.004**	**0.025**	-0.017	0.224	0.023	0.148	-0.012	0.191	0.005	0.408
Log insulin	0.615	0.007	-0.008	0.912	0.073	0.445	-0.048	0.325	-0.062	0.327

BMI, BMI-z-score; HDL, High-density lipoprotein cholesterol; LDL, Low-density lipoprotein cholesterol; LPA, light-intensity physical activity; MPA, moderate-intensity physical activity; PA, physical activity; ST, sedentary time; VPA, vigorous-intensity physical activity.

Model p-value and R-square are based on the unadjusted model. Regression coefficients and corresponding p-values were adjusted for age, sex, race/ethnicity, socioeconomic status, smoking, sodium, saturated fat, total calories.

Regression coefficients correspond to change in the log-ratio of the given behavior relative to other behaviors.

Statistically significant associations **(p<0.05)** are highlighted in bold.

^a^ Association was no longer significant after adjusting for log waist circumference.

For the individual behaviors, after adjustments for covariates, ST relative to the other three behaviors had significant positive associations with log waist circumference (γ_ST_ = 0.029; p = 0.013) and diastolic blood pressure (γ_ST_ = 2.700; p = 0.018). Time spent in LPA relative to the other three behaviors had a significant negative association with diastolic blood pressure (γ_LPA_ = -2.892; p = 0.026). Time spent in MPA relative to the other three behaviors had a significant negative association with HDL-cholesterol; however, this association was no longer significant after adjusting for waist circumference. Time spent in VPA relative to the other three behaviors had significant negative associations with BMI z-score (γ_VPA_ = -0.206; p = 0.005) and log waist circumference (γ_VPA_ = -0.033; p = 0.001), and significant positive associations with diastolic blood pressure (γ_VPA_ = 1.246; p = 0.038) and HDL-cholesterol (γ_VPA_ = 0.058; p = <0.001). No other significant associations were observed.

## Discussion

The objectives of this paper were to use compositional analyses to examine the combined and relative associations of ST, LPA, MPA, VPA with cardiometabolic biomarkers in a representative sample of 6–17 year-olds living in the United States. The composition of ST and the various intensities of PA were significantly associated with all cardiometabolic biomarkers in the full analytical sample, except for CRP. Therefore, the combined associations of ST, LPA, MPA, and VPA during the waking day may be important for cardiometabolic health. In terms of relative associations, more time spent in VPA relative to the other behaviors was favorable for adiposity indicators and HDL-cholesterol. In contrast, more time spent in ST relative to other behaviors was unfavorable for waist circumference and diastolic blood pressure. Few associations were observed for LPA and MPA. Additionally, almost no associations were observed in the fasting sub-sample.

Compositional analysis is a novel method that has only been used in a handful of other studies examining collective and relative behavioral associations with adiposity and other cardiometabolic biomarkers in children and youth.[8–10] For instance, in a large representative sample of Canadian 6–17 year-olds, the composition of sleep, ST, LPA, and MVPA were significantly associated with all cardiometabolic biomarkers examined including indicators of adiposity, blood pressure, blood lipids, CRP, and insulin.[9] In general, significant favorable associations were observed for MVPA and sleep, relative to other behaviors, whereas significant unfavorable associations were observed for ST and LPA.[9] Similar findings were observed for adiposity indicators in a large international sample of 9–11 year olds[8] and a smaller sample of children aged 10–13 years from Kingston, Canada.[10] However, significant associations were not observed for ST and sleep, relative to other behaviors in the smaller Canadian sample.[10]

Compared to previous studies in children and youth using compositional analyses, the present study focused on the waking day. Only two other studies to our knowledge have focused on a subdivision of the 24-hour data when using this method.[12] Similar to the present study in terms of waist circumference, ST was positively associated and VPA was negatively associated with BMI z-score in 420 adolescents aged 12–19 years from the Czech Republic.[29] Additionally, the composition of ST, LPA, and MVPA during the school day was significantly associated with adiposity indicators in a sample of 318 children aged 10–11 years from the United Kingdom.[12] These combined cross-sectional findings from all studies using compositional analyses support an integrated approach for health promotion in children and youth compared to focusing on movement behaviors in isolation. Future longitudinal and experimental studies are needed to confirm and build on this cross-sectional evidence. Additionally, future research is needed to examine whether variables such as age and sex moderate the compositional associations with cardiometabolic health.

A unique aspect of the present study, compared to most other studies using compositional analyses, is MPA and VPA were considered as separate behaviors, instead of being combined into MVPA. Though compositional geometric mean bar plots showed a similar pattern for MPA and VPA, significant relative associations with adiposity indicators were only observed for VPA and not MPA in regression models. Additionally, VPA associations were in the favorable direction, whereas the opposite was observed for LPA and ST. A systematic review of studies that have used traditional approaches to examine associations between PA, adiposity, and other cardiometabolic biomarkers, observed more consistent and stronger associations with higher intensity PA (e.g., VPA) compared to lower intensity PA (e.g., LPA, MPA), in particular for adiposity.[1] Since the present study examined relative associations that took into account the time spent in all waking day movement behaviors, study findings strengthen the evidence base in this area.

Though some general patterns were observed that suggested compositions with more time in higher-intensity activities may be optimal for some aspects of cardiometabolic health, a number of null associations and some inconsistent associations were also observed. In particular, almost no associations were observed with fasting lipids (LDL-cholesterol, triglycerides), insulin, and glucose measures. This is in contrast to the previous study on a representative sample of Canadian children and youth where a number of associations were observed across cardiometabolic biomarkers using compositional analyses.[9] Some methodological differences exist between the Canadian and present study, with one notable difference being the inclusion of sleep in the Canadian sample. A number of interactions have been observed between sleep and sedentary behavior and between sleep and PA in children and youth.[30] Sleep also has important links with cardiometabolic health.[31] Therefore, it seems important for future studies to consider 24-hour accelerometer wear protocols so the health impact of 24-hour compositions and relevant subdivisions can be examined.

Main strengths of this study include the representative sample, the compositional analyses that took into account the co-dependent nature of the ST and PA data, the objective measures of ST and PA, and the range of cardiometabolic biomarkers. A main limitation of the study is the cross-sectional data, which prevents casual inferences from being made. Data on sleep duration were also not available for this age group. Additionally, in order to conduct the analyses some daily zero values, mainly for VPA, were replaced with 0.5 minutes. It is important to note that previous research has shown that VPA is significantly lower when using 60 second epochs, as was used in NHANES, compared to 30, 15, and 5 second epochs in both children and adolescents. [32] Though accelerometer cut-points are useful for categorizing the data into different intensities, novel analytical techniques to identify patterns of behaviors, such as machine learning, [33] may provide new insights into the collective and relative associations between movement behaviors and cardiometabolic health. Finally, though we adjusted for a number of potential confounders, residual confounding cannot be ruled out.

## Conclusions

Compositional analysis is an appropriate method for examining collective and relative associations between waking day movement behaviors and cardiometabolic biomarkers. The composition of ST, LPA, MPA, and VPA in this large representative sample of children and youth from the United States was significantly associated with many aspects of cardiometabolic health. Findings from this study also suggest that compositions with more time in higher-intensity activities may be better for some aspects of cardiometabolic health, such as adiposity. This study contributes to a growing body of evidence that supports an integrated approach to movement behaviors for health promotion in the pediatric population. Future longitudinal and experimental studies are needed to build on these findings as well as research that examines potential effect modifiers of these associations.
